# Extracellular matrix stiffness modulates angiogenic properties of the retinal pigment epithelium

**DOI:** 10.1038/s41598-025-27140-4

**Published:** 2025-11-18

**Authors:** Lasse Wolfram, Melanie Schwämmle, Clara Gimpel, David A. Merle, Jiaqi Tang, Simon J. Clark, Daniel Böhringer, Günther Schlunck

**Affiliations:** 1 Eye Center, Medical Center - Faculty of Medicine, University of Freiburg, Freiburg, Germany; 2 Department for Ophthalmology, Institute for Ophthalmic Research, Eberhard Karls University of Tübingen, Tübingen, Germany; 3 Department for Ophthalmology, University Eye Clinic, Eberhard Karls University of Tübingen, Tübingen, Germany; 4 Faculty of Biology, University of Freiburg, Freiburg, Germany; 5 Department of Neurology, Schlosspark-Klinik Charlottenburg, Berlin, Germany; 6 Lydia Becker Institute of Immunology and Inflammation, University of Manchester, Manchester, UK

**Keywords:** Extracellular matrix (ECM), Bruch’s membrane (BrM), Retinal pigment epithelium (RPE), Age-related macular degeneration (AMD), Tissue stiffness, Angiogenesis, Retinal diseases, Extracellular matrix

## Abstract

Physiological and pathological processes, such as aging and basal deposit aggregation in degenerative retinal diseases like age-related macular degeneration (AMD), alter the mechanical properties of Bruch’s membrane (BrM). These mechanical changes in the extracellular matrix (ECM) significantly affect retinal pigment epithelial (RPE) cells, influencing their morphology, transcriptome and angiogenic behavior. ARPE-19 cells were cultured on hydrogels of physiological stiffness (30 and 80 kPa) and on conventional tissue culture plastic (TCP) for comparison. Gene expression was analyzed by droplet digital PCR (ddPCR), while protein-level changes were examined using immunofluorescence (IF), Western blotting (WB) and enzyme-linked immunosorbent assays (ELISA). Stiffness-dependent modulation of endothelial cells by RPE-conditioned media was investigated using in vitro angiogenesis assays. RPE cells cultured on softer substrates exhibited enhanced angiogenic properties, including increased expression of CD44 and vascular endothelial growth factor (VEGF). In contrast, stiffer substrates promoted antiangiogenic responses, associated with altered distribution of thrombospondin 1 (THBS1). These findings underscore the importance of ECM mechanics in modulating angiogenic signaling and highlight their potential relevance in retinal pathologies such as AMD. Local disruptions in adhesion or mechanical cues, potentially caused by basal deposits, may contribute to proangiogenic behavior even in the context of globally increased tissue stiffness with age.

**Supplementary Information:**

The online version contains supplementary material available at 10.1038/s41598-025-27140-4.

## Introduction

Age-related macular degeneration (AMD) is the leading cause of irreversible blindness in individuals over the age of 55 worldwide^1^. Due to the complex interplay of genetic risks, natural aging and lifestyle factors, AMD is classified as a multifactorial disease^2^. Clinically, AMD manifests in two primary forms: the dry (atrophic) form, which can progress to geographic atrophy (GA) of retinal pigment epithelial (RPE) cells with subsequent retinal decay, and the wet (neovascular) form (nAMD), characterized by macular neovascularization (MNV) that leads to retinal edema, subretinal bleeding, or scar formation. Although these forms have distinct pathological mechanisms, advanced dry AMD can evolve into the neovascular form in some patients^3^.

Angiogenesis, defined as the biological process of blood vessel development from an existing vascular bed, is an integral component of various physiological processes, including body growth, development and repair^4^. In this process, lysis of the basal membrane and subsequent proliferation, migration and sprouting of endothelial cells from existing blood vessels play a pivotal role^5^. Vascular endothelial growth factor (VEGF) is among the most potent pro-angiogenic factors^6^. Current treatments for nAMD therefore have largely adopted anti-VEGF therapies, which are remarkably effective in stabilizing or even improving visual acuity in a substantial fraction of patients. However, 25–50% of patients suffer from persistent disease activity manifested as edema, bleeding or fibrosis, despite VEGF inhibitor treatment^7^. The recent emergence of bispecific antibodies such as Faricimab, targeting both VEGF and angiopoietin 2 (Ang-2), offers a promising avenue for enhanced therapy. Dual inhibition agents such as Faricimab target multiple pathways involved in the pathogenesis of nAMD, potentially enhancing disease management and extending treatment intervals by modulating a broader range of angiomodulatory factors.^8–10^

Angiogenesis is tightly regulated by a balance of pro- and anti-angiogenic factors, influenced by extracellular matrix (ECM) composition and structure^4^. Disruptions in this balance can lead to pathological neovascularization such as macular neovascularization (MNV) observed in neovascular AMD and other conditions, including proliferative diabetic retinopathy^4,6,11^. While these factors share roles in angiogenesis and cell-ECM interactions, they differ in mechanisms, expression patterns and concentration-dependent effects. A deeper understanding of their interplay is essential for identifying novel therapeutic targets in ocular vascular disorders.

Based on manifold mechanotransduction processes, cells perceive the biomechanical properties of the ECM and are directly influenced by its changes, affecting various cellular processes including proliferation, differentiation, migration and apoptosis^12–17^. Common age-related diseases, such as atherosclerosis, are associated with a loss of function of elastic connective tissue components and increasing tissue stiffness^18^. Several ocular diseases, including AMD and primary open-angle glaucoma, are accompanied by changes in tissue stiffness^19,20^. The deposition of extracellular material, manifesting as linear and laminar deposits or drusen, is associated with alterations in the biomechanical properties of Bruch’s membrane (BrM), as evidenced in BrMs of advanced age^21,22^.

Mechanical properties of the ECM, including stiffness, are known to influence angiogenesis^23^; however, the interplay between mechanical cues and the regulation of pro- and anti-angiogenic signaling, particularly in ocular cells, remains incompletely understood. Notably, conventional tissue culture plastic (TCP) exhibits a stiffness of approximately 3 GPa (3,000,000 kPa)^24^, which exceeds physiological tissue stiffness (typically 1–80 kPa) by several orders of magnitude^16^. It is therefore considered essential to account for proper BrM/RPE crosstalk when developing model systems for AMD disease^25^.

As recently shown by our group, substrate stiffness modulates the expression of both messenger RNAs (mRNAs) and small RNAs (sRNAs) such as microRNAs (miRNAs) in RPE cells, with miRNA changes likely contributing to tissue homeostasis by fine-tuning a broad range of intracellular pathways^26^. Gene set enrichment analysis further suggested that substrate stiffness differentially regulates angiogenesis-related genes, with increased expression of factors such as *ALDH1A3*, *CTGF*, *PEDF* and *THBS1* on stiff substrates and *CD44*, *MT2A*, *NOV*, *PAI-1* and *VEGF* on soft substrates.

In this study, we investigated the influence of substrate stiffness on the expression of pro- and anti-angiogenic factors, revealing enhanced angiogenic properties of RPE cells on softer substrates.

## Methods

### Cell culture of ARPE-19 and HUVEC

ARPE-19 cells (American Type Culture Collection) were cultured in DMEM/Ham’s F-12 media supplemented with 10% FBS, 1% L-Glutamine (200mM), 1% Penicillin-Streptomycin (Biochrom, Berlin, Germany), 1% MEM Non-Essential Amino Acids Solution, 1% Insulin-Transferrin-Selenium (Thermo Fisher Scientific, Waltham, MA, USA) and 1% HEPES buffer (1 M) (PAN-Biotech, Aidenbach, Germany). Only early passages of cells (*P* < 10) were used for experiments. For stiffness experiments, PA gels of 30 kPa and 80 kPa were prepared using acrylamide and bis-acrylamide solutions with substrate stiffness controlled by the proportion of bis-acrylamide and verified by bio-indentation, as previously described^26^. These gels recapitulate physiological tissue stiffness (approximately 1–80 kPa)^16^, while tissue culture plastic (TCP, ~ 3 GPa)^24^ served as non-physiological control. Adapted ARPE-19 cell numbers were used to achieve comparable confluence: 1,200,000 cells on 30 kPa gels, 800,000 on 80 kPa gels and 500,000 on TCP^26^.

Human umbilical vein endothelial cells (HUVECs) (Lonza, Basel, Switzerland, Lot Number C2519A) were cultured in Endothelial Cell Growth Medium (ECGM) (Lonza, Basel Switzerland) and passaged before reaching confluence. Only early passages of confluent cells (*P* < 6) were used to maintain endothelial properties. For all angiogenesis assays, HUVECs were stimulated with ARPE-19-conditioned medium (CM). To generate CM, ARPE-19 cells were grown on different substrates and the medium was changed to 1% FBS one day before collection to minimize FBS-related growth effects.

### Angiogenesis assays

The MTT (3-[4,5-dimethylthiazol-2-yl]-2,5 diphenyl tetrazolium bromide)-based proliferation assay^27,28^ was performed by culturing HUVECs at an initial density of 4000 cells/well in a 96-well plate for 72 h. Four hours after seeding, ECGM was replaced with Endothelial Cell Basal Medium (ECBM) (Lonza, Basel Switzerland) containing 2% FBS to reduce background stimulation. The following day, cells were stimulated with ARPE-19-CM in eight replicates per condition. ECGM and unconditioned ARPE-19 medium served as positive and negative controls, respectively.

The Migration assay^29^ was conducted in individual chambers using silicone dividers placed in 24-well plates (ibidi, Gräfelfing, Germany). 30,000 HUVECs in 70 µl ECGM were seeded in each chamber. After two hours, ECGM was replaced with ECBM. The following day, silicon dividers were removed to create cell-free gaps of 500 μm width. HUVECs were then stimulated with ARPE-19-CM in three to four replicates per condition. ECGM and unconditioned ARPE-19 medium served as positive and negative controls, respectively. Cell migration into the gap areas was monitored by phase contrast microscopy. Images were taken at 0, 4, 8 and 12 h after stimulation. Cell-free areas were quantified using the MRI Wound Healing macro for ImageJ^30^.

The Spheroid sprouting assay^29^ was performed using collagen-embedded HUVECs. Spheroids were generated by suspending HUVECs in 20% methylcellulose-ECBM. Each spheroid contained 500 HUVECs per 25 µl drop of methylcellulose-ECBM. The following day, the spheroids were washed with PBS and centrifuged. The supernatant was discarded and the spheroids were resuspended in methylcellulose and FBS. For collagen embedding, Collagen Type I (Corning, NY, USA) and Media 199 10x (Sigma-Aldrich, Merck, Darmstadt, Germany) were mixed and titrated to pH = 7,0, using NaOH before adding HEPES buffer to the gel solution. A preheated 24-well plate, each containing 0,5 ml of collagen gel solution with approximately 50 spheroids, was used for stimulation with ARPE-19-CM. VEGF and unconditioned ARPE-19 medium served as positive and negative controls, respectively. Spheroids were observed using phase-contrast microscopy and cumulative lengths of sprouts were analyzed after 16 h in culture using ImageJ.

### RNA isolation and protein isolation

RNAs and proteins were isolated after three weeks of confluent cultivation as previously described^26^. Isolation of mRNAs and sRNAs was performed using the Qiagen miRNeasy and RNeasy Kits (Qiagen, Venlo, The Netherlands). RNA quality was assessed using a Bioanalyzer (Agilent, Santa Clara, CA, USA) and the total RNA concentration was determined using a NanoDrop ND-1000 Spectrophotometer (Peqlab Biotechnologie, VWR, Radnor, PA, USA).

Proteins were extracted by lysing cells in lysis buffer containing TRIS [50 mM] (Sigma-Aldrich, Merck, Darmstadt, Germany), EDTA [1 mM] (Serva Electrophoresis, Heidelberg, Germany), 1% Triton X-100 (Sigma-Aldrich, Merck, Darmstadt, Germany), protease inhibitors (cOmplete Mini EDTA-free Protease-Inhibitor-Cocktail, 04693159001, F. Hoffmann-La Roche, Basel, Switzerland) and phosphatase inhibitors (PhosSTOP, 04906845001, F. Hoffmann-La Roche, Basel, Switzerland). Total protein content was determined by bicinchoninic acid (BCA) assay (Pierce BCA Protein Assay Kit, Thermo Scientific, Thermo Fisher Scientific, Waltham, MA, USA). cDNA synthesis using the SuperScript IV Reverse Transcriptase Kit (Thermo Fisher Scientific, Waltham, MA, USA) was performed using normalized amounts of material.

### Droplet digital PCR (ddPCR)

Droplet digital PCR (ddPCR) analysis was performed using the QX 200 Droplet Digital PCR system (Bio-Rad Laboratories, Hercules, CA, USA) with TaqMan Gene Expression Assays (Thermo Fisher Scientific, Waltham, MA, USA) to quantify mRNA expression levels of angiogenesis-related genes including *ALDH1A3*, *CD44*, *CTGF*, *MT2A*, *NOV*, *PAI-1*, *PEDF*, *THBS1* and *VEGF*.

### Western blot (WB) analysis

CD44 protein expression was analyzed by Western blotting (WB) using CD44 (E7K2Y) XP^®^ Rabbit mAb (37259 S, Cell Signaling Technology, Danvers, MA, USA) as primary antibody. CD44 is a cell surface glycoprotein involved in cell adhesion, migration and proliferation that serves as a receptor for hyaluronic acid and other extracellular matrix components. GAPDH served as loading control using Anti-Glyceraldehyde-3-Phosphate Dehydrogenase Antibody, clone 6C5 (MAB374, Sigma-Aldrich, Burlington, MA, USA). Secondary antibodies used were Peroxidase AffiniPure Goat Anti-Rabbit IgG (H + L) (111-035-003, Jackson ImmunoResearch Laboratories, West Grove, PA, USA) and Peroxidase AffiniPure Goat Anti-Mouse IgG (H + L) (115-035-003, Jackson ImmunoResearch Laboratories, West Grove, PA, USA). Western blotting was performed using the Mini-Protean wet electroblotting system (Bio-Rad Laboratories, Hercules, CA, USA) with normalized amounts of material. Semiquantitative analysis of normalized differences in gray values was conducted using ImageJ.

### Enzyme-linked immunosorbent assay (ELISA)

VEGF protein concentration in ARPE-19-CM was quantified using an enzyme-linked immunosorbent assay (LEGEND MAX Human VEGF ELISA Kit, 446507, BioLegend, San Diego, CA, USA) according to the manufacturer’s instructions. VEGF is a key pro-angiogenic cytokine that promotes endothelial cell proliferation, migration and tube formation.

### Immunofluorescence (IF) staining and microscopy

Immunofluorescence (IF) staining was performed to analyze protein localization and expression patterns. Cells were fixed and stained using the following reagents: 4′,6-diamidino-2-phenylindole, dihydrochloride (10374168, Thermo Fisher Scientific, Waltham, MA, USA) for nuclear staining; phalloidin-tetramethylrhodamine B isothiocyanate (P1951, Sigma-Aldrich, Merck, Darmstadt, Germany) for labeling the actin cytoskeleton. Primary antibodies included Thrombospondin 1 Monoclonal Antibody (A6.1) (MA5-13398, Thermo Fisher Scientific, Waltham, MA, USA) to detect THBS1, an anti-angiogenic matricellular protein, and ZO-1 Polyclonal Antibody (40-2200, Thermo Fisher Scientific, Waltham, MA, USA) to visualize tight junction integrity. Secondary antibodies used were Alexa Fluor 488 AffiniPure Goat Anti-Mouse IgG (H + L) (115-545-146, Jackson ImmunoResearch Laboratories, West Grove, PA, USA) and Cy5 AffiniPure Donkey Anti-Rabbit IgG (H + L) (711-175-152, Jackson ImmunoResearch Laboratories, West Grove, PA, USA).

PA gels of varying substrate stiffness were cast and coated as previously described^26^ and, together with fibronectin-coated coverslips, were used with adapted ARPE-19 cell numbers, as published earlier^26^: 120,000 cells on PA gels of 30 kPa, 80,000 cells on PA gels of 80 kPa and 50,000 cells on TCP. After three weeks in confluent culture, IF staining and visualization were performed using a TCS-SP 8 confocal microscope (Leica, Wetzlar, Germany).

### Statistics and data visualization

Data analysis and visualization were conducted using GraphPad Prism 8 (GraphPad Software, Insight Partners, New York City, NY, USA), with statistical tests as indicated in the figure legends. Image processing was performed using ImageJ 2.52u^31^, Inkscape 1.0.1 ^32^ and Gimp 2.10 ^33^.

### Ethics statement

All experiments were conducted in accordance with relevant guidelines and regulations. The study did not involve human participants or primary human tissues. Human retinal pigment epithelial cells (ARPE-19) and human umbilical vein endothelial cells (HUVECs) were obtained from commercial suppliers (ATCC and Lonza, respectively) and used in compliance with institutional biosafety regulations. As only established, commercially available cell lines were used, ethical approval was not required.

## Results

### Stiffness-dependent angiogenesis-related gene expression

**Fig. 1:**
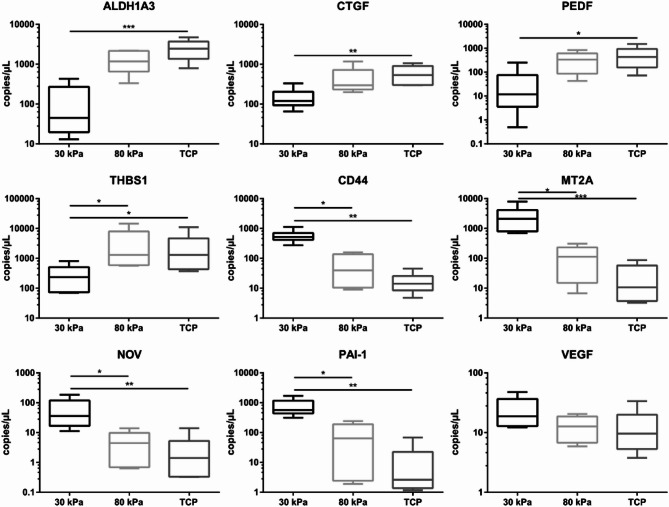
Summary of ddPCR data from six individual experiments (*n* = 6), visualized as logarithmically scaled box plots. Overall statistical significance was assessed using the Kruskal-Wallis test and pairwise comparisons indicated by asterisks were determined using post-hoc Dunn test (*: *p* < 0.05; **: *p* < 0.01; ***: *p* < 0.001).

Substrate stiffness has been shown to modulate cellular behavior and gene expression in various cell types, including RPE cells^26^. To investigate whether mechanical properties influence angiogenesis-related gene expression in ARPE-19 cells, we analyzed key pro- and anti-angiogenic factors using ddPCR. ddPCR analysis from six independent experiments (*n* = 6) confirmed a stiffness-dependent regulation of angiogenesis-related genes, with higher expression of *ALDH1A3*, *CTGF*, *PEDF* and *THBS1* on stiff substrates and increased levels of *CD44*, *MT2A*, *NOV*, *PAI-1* and *VEGF* on soft substrates (Fig. 1). These findings demonstrate that substrate stiffness significantly modulates the expression profile of angiogenesis-related genes, with softer substrates promoting pro-angiogenic gene expression while stiffer substrates favor anti-angiogenic gene expression.

### Stiffness-dependent expression of angiogenic modulators

**Fig. 2:**
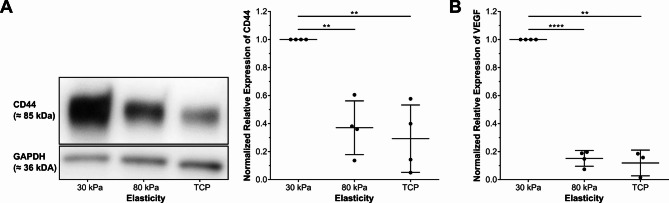
Stiffness-dependent expression of angiogenic factors in ARPE-19 cells grown on substrates with stiffnesses of 30 kPa, 80 kPa and TCP. (**A**) Representative WB of CD44 and semiquantitative analysis from *n* = 4 independent experiments, visualizing normalized expression of CD44 based on GAPDH as the endogenous control, with error bars depicting standard deviation. The full WBs are available in Supplementary Figures S1 and S2 online. (**B**) Normalized relative expression of VEGF in ARPE-19-CM (*n* = 4; TCP: *n* = 3). VEGF levels were normalized to GAPDH concentrations, with media background subtracted. Data are presented as mean relative expression compared to 30 kPa ± SD with individual data points shown. Statistical significance was assessed using one-sample t-tests when comparing normalized values to a hypothetical value of 1.00 (ns: non-significant; *: *p* < 0.05; **: *p* < 0.01; ***: *p* < 0.001).

To validate our gene expression findings at the protein level and confirm functional relevance, we examined key angiogenic modulators identified in the ddPCR analysis using WB and ELISA approaches^4^. To further explore the effect of stiffness on the expression of angiogenic modulators on the protein level, we conducted WB, ELISA or IF staining. CD44, as shown by WB (Fig. 2A), and VEGF, as shown by ELISA (Fig. 2B), exhibited significantly decreased protein expression with increasing substrate stiffness. These findings were consistent with the ddPCR results (Fig. 1). These protein-level analyses confirm that substrate stiffness influences not only gene expression but also protein abundance of key angiogenic factors, supporting the functional relevance of our transcriptional findings.

### Stiffness-dependent localization of angiogenic modulators

**Fig. 3:**
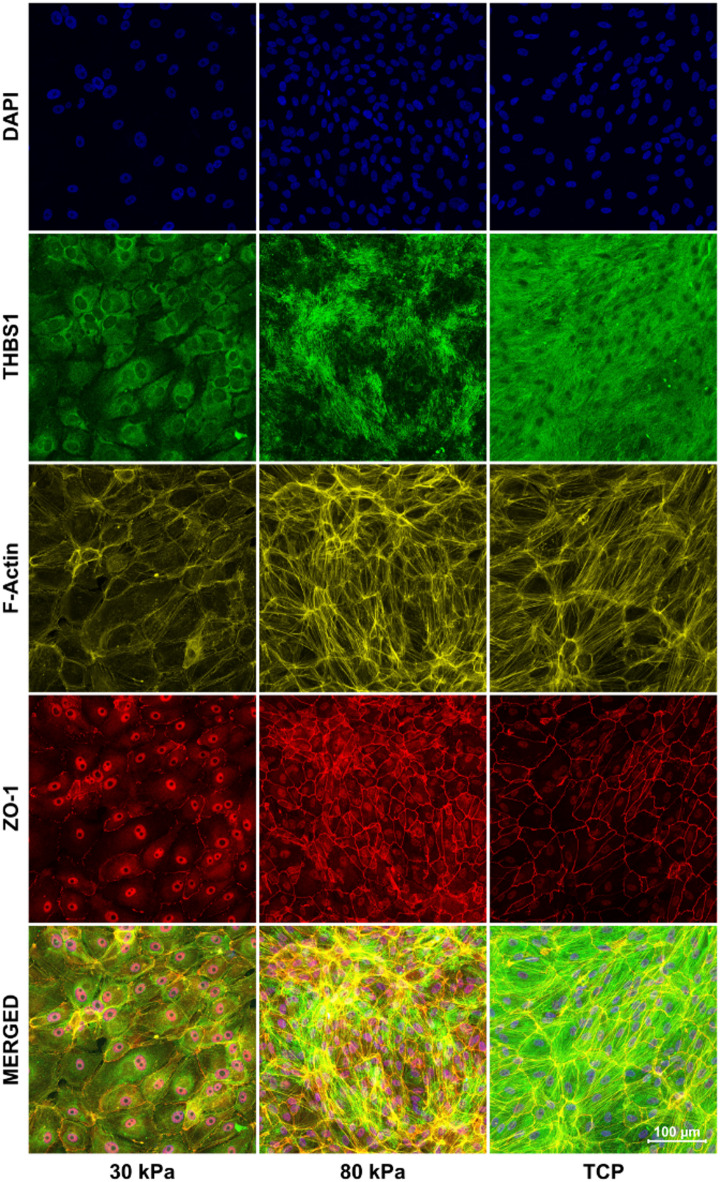
Stiffness-dependent localization of THBS1 in ARPE-19 cells grown on substrates with stiffnesses of 30 kPa, 80 kPa and TCP. IF images illustrating the localization of the nuclei/DNA (DAPI, blue), THBS1 (green), F-Actin (yellow), cell borders (ZO-1, red) and merged images. The images are represented as orthogonal projections created from a confocal stack of 17 individual images, providing a comprehensive view of the cells from their apical to basal extent.

Beyond changes in expression levels, substrate stiffness can also influence protein localization and cellular organization, which may affect protein function and cell-cell interactions^12–17^. We therefore examined the subcellular distribution of key proteins using immunofluorescence microscopy. THBS1 exhibited a stiffness-dependent distribution pattern in IF staining (Fig. 3). On soft substrates, the protein localized predominantly to the perinuclear cytoplasm, whereas on stiff substrates and TCP, it formed distinct fibrous-like extracellular deposits. The overall signal intensity of the anti-angiogenic THBS1 was markedly increased on stiffer substrates, consistent with ddPCR data (Fig. 1). In addition, RPE cells cultured on softer substrates appeared fewer in number but larger in size, as indicated by DNA (DAPI) and ZO-1 staining, while stiffer substrates promoted the formation of denser, more organized F-actin fibers. These results indicate that substrate stiffness not only affects protein expression but also influences subcellular protein localization and overall cellular morphology, potentially modulating protein function and cell-matrix interactions.

### Stiffness-dependent angiomodulatory properties

**Fig. 4:**
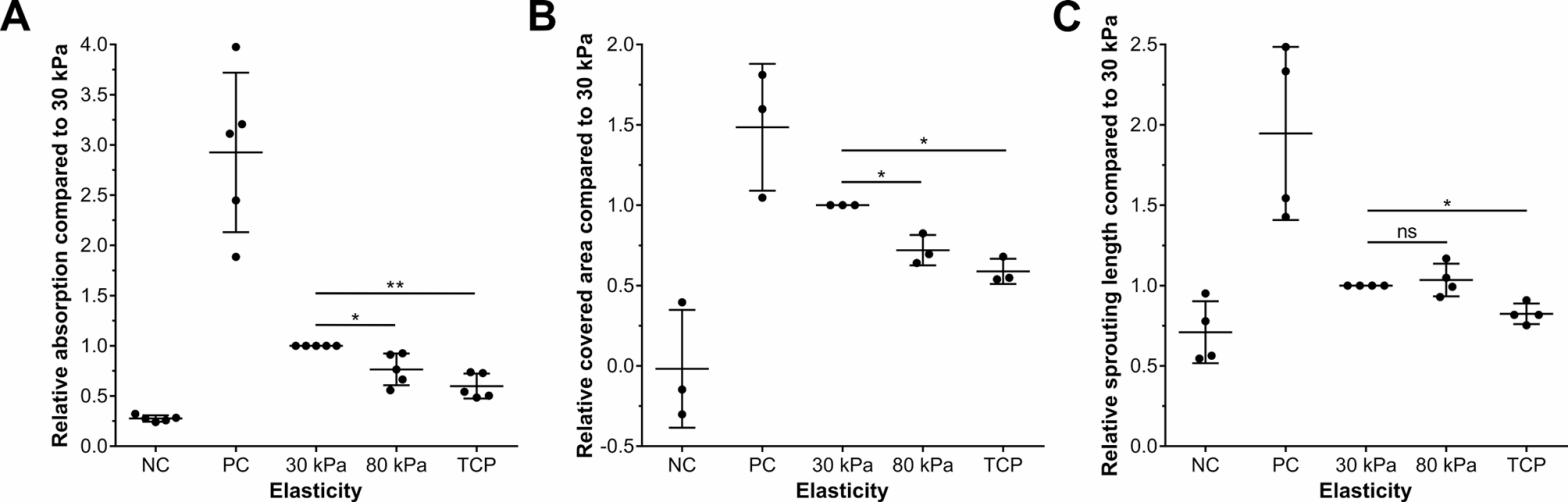
Effects of media conditioned by ARPE-19-cells on different cell culture substrates on HUVECs in proliferation, migration and spheroid sprouting assays. (**A**) MTT-based proliferation assay, representing the average values from five individual experiments (*n* = 5). The graph depicts the relative absorption compared to 30 kPa after 72 h of incubation. ECGM and unconditioned ARPE-19 medium served as positive (PC) and negative (NC) controls, respectively. (**B**) Migration assay, depicting data from three separate experiments (*n* = 3). The graph shows the relative area covered by endothelial cells compared to 30 kPa twelve-hours after seeding. ECGM and unconditioned ARPE-19 medium served as positive (PC) and negative (NC) controls, respectively. (**C**) Spheroid sprouting assay, integrating data derived from four individual experiments (*n* = 4). The graph represents the relative cumulative lengths of sprouts compared to 30 kPa, measured 16 h after stimulation. VEGF and unconditioned ARPE-19 medium served as positive (PC) and negative (NC) controls, respectively. Data are presented as mean relative expression compared to 30 kPa ± SD with individual data points shown. Statistical significance in all three angiogenesis assays was assessed using one-sample t-tests when comparing normalized values to a hypothetical value of 1.00 (ns: non-significant; *: *p* < 0.05; **: *p* < 0.01; ***: *p* < 0.001).

The ultimate test of angiogenic potential lies in the functional response of endothelial cells to secreted factors. CM from cells grown under different mechanical conditions may exhibit distinct angiogenic properties due to the differential expression of pro- and anti-angiogenic factors^4,23^. To assess the functional consequences of stiffness-dependent gene and protein expression changes, we evaluated the angiogenic potential of conditioned media using established endothelial cell assays. Using angiogenesis assays, we analyzed endothelial cell proliferation, migration and sprouting in response to CM of ARPE-19 cells cultured on different substrates. The MTT assay revealed a significant decrease in absorption, indicating reduced proliferation with increasing substrate stiffness (Fig. 4A). The migration assay likewise showed significantly less promigratory factor expression by ARPE-19 on stiffer substrates (Fig. 4B). Consistently, the spheroid sprouting assay also demonstrated a reduction in sprout length with increasing stiffness of the ARPE-19 substrate for media conditioning (Fig. 4C). These functional assays demonstrate that substrate stiffness influences the angiogenic properties of ARPE-19-CM, with softer substrates promoting endothelial cell proliferation, migration and sprouting capacity.

## Discussion

Our findings highlight a substrate-dependent regulation of key angiogenic modulators in ARPE-19 cells, with pro-angiogenic factors such as *VEGF*, *CD44*, *NOV* and *PAI-1* showing increased expression on softer substrates, while anti-angiogenic factors, including *THBS1*, *PEDF* and *CTGF* were more abundant on stiffer substrates. This mechanobiological regulation of angiogenic balance represents a novel finding that extends our understanding of RPE cell biology beyond traditional biochemical signaling pathways.The increased VEGF protein levels in RPE cells cultured on softer substrates, combined with higher mRNA expression trends, align with previous observations that mechanical properties influence growth factor secretion in various cell types. CD44, a key regulator of cell-ECM interactions and a co-receptor for receptor tyrosine kinase signaling^34^, exhibited higher expression on softer substrates, supporting its established role in angiogenesis and MNV formation^35–38^. The coordinate upregulation of *NOV*, a matricellular protein involved in integrin-mediated cell adhesion and migration, which has been implicated in promoting angiogenesis^39,40^, and *PAI-1*, known for its dual role in modulating angiogenesis in a concentration-dependent manner^41,42^, under softer conditions, suggests a comprehensive pro-angiogenic shift driven by mechanical cues.

Conversely, the preferential expression of anti-angiogenic factors on stiffer substrates may represent a protective mechanism. THBS1, a potent inhibitor of neovascularization with known relevance to AMD pathology^43–46^, showed both increased expression and enhanced extracellular deposition on stiffer substrates, suggesting strengthened anti-angiogenic barriers under these conditions. The clinical relevance of this finding is underscored by observations that THBS1 is highly expressed in BrM but markedly reduced in AMD patients, particularly in the BrM-choroid complex and areas of MNV^47^. Similarly, the reduced expression of *PEDF*, known for its neuroprotective and anti-angiogenic effects in retinal diseases^48–51^, and *CTGF*, primarily linked to fibrotic processes but also involved in proliferation, adhesion and angiogenesis, with the potential to inhibit VEGF-induced angiogenesis^52,53^, on softer substrates may compromise the natural anti-angiogenic defense mechanisms of the RPE.The functional validation through angiogenesis assays demonstrated that these molecular changes translate to altered endothelial cell behavior. The changes toward enhanced proliferation, migration and sprouting capacity in response to CM from RPE cells grown on softer substrates provide functional evidence for the biological significance of stiffness-dependent angiogenic factor regulation. These findings complement recent studies highlighting the impact of mechanical cues on angiogenesis pathways^23^ and align with reports suggesting that stiffer environments tend to favor anti-angiogenic responses while softer substrates promote pro-angiogenic signaling^54,55^. Our results are consistent with the work of Zhang and colleagues, who demonstrated alterations in key pathways in RPE cells cultured on amniotic membranes (stiffness: E = 1.22–5.50 kPa) compared to those on Matrigel-coated TCP (stiffness: E = 70.72-175.93 MPa)^55^, showing significant substrate-dependent changes in angiogenesis-related pathways. Although direct comparisons between absolute E-moduli measured by atomic force microscopy (AFM) or nanoindentation are not feasible as the values depend on parameters such as loading rates, which are not standardized and vary between studies, a relative comparison between soft substrates and TCP remains valid. This cross-validation across different experimental systems strengthens the evidence for mechanical regulation of RPE angiogenic function.

The apparent paradox between aging-associated increases in ocular tissue stiffness^19,20^ and our observation that softer substrates promote pro-angiogenic environments can be reconciled by considering the local microenvironment of the RPE. Softer cell culture substrates reduce integrin-dependent cell-matrix adhesion by limiting cellular traction forces, leading to smaller focal adhesions, weaker actin cytoskeleton and diminished downstream signaling (e.g., FAK, ILK, ERK)^12,13^. Similarly, basal laminar and linear deposits in AMD may impair RPE adhesion due to altered composition and reduced local stiffness and/or a decreased availability of integrin-binding sites. These deposits could thus mimic the effects of soft substrates by promoting RPE deadhesion, despite overall increase in tissue stiffness with age. This mechanistic framework suggests that AMD pathogenesis involves not only biochemical changes but also mechanical disruption of normal RPE-ECM crosstalk, potentially explaining choroidal neovascularization development even in the context of tissue stiffening. Such localized changes are unlikely to be captured by bulk or AFM stiffness measurements, especially given the thinness of relevant basal layers and technical challenges such as sample mounting. Therefore, increased overall tissue stiffness with age does not necessarily contradict focal adhesion loss at the RPE-Bruch’s membrane interface. Instead, it underscores the importance of characterizing the mechanical microenvironment of the RPE at high spatial resolution, particularly in the context of basal laminar and linear deposits and their potential to disrupt integrin-mediated adhesion.

Several methodological considerations merit discussion. ARPE-19 cells were cultured for three weeks to promote differentiation and RPE-like morphology, following established approaches^56^. Despite this optimization, inherent limitations of this immortalized cell line compared to primary RPE cells must be acknowledged. HUVECs were used as a robust and reproducible model to assess paracrine angiogenic effects, as stiffness-dependent vascular responses are conserved across endothelial subtypes^57,58^. While this approach provides valuable insights into RPE angiogenic potential under varying mechanical conditions, future studies employing ocular endothelial cells will be important for enhanced translational relevance.

This study establishes substrate stiffness as a fundamental regulator of angiogenic factor expression in RPE cells, providing new insights into the mechanobiological basis of retinal angiogenic diseases. The findings suggest that mechanical factors may contribute to AMD pathogenesis through disruption of normal RPE mechanotransduction, highlighting the importance of considering ECM properties in both disease understanding and therapeutic development. While clinical applications remain distant, this work emphasizes the need for physiologically relevant culture systems in retinal disease research and points toward potential mechanical targets for future therapeutic intervention beyond traditional anti-VEGF approaches.

## Supplementary Information

Below is the link to the electronic supplementary material.

Supplementary Material 1

## Data Availability

The datasets analyzed during the current study are available in the Gene Expression Omnibus (GEO) repository, [https://www.ncbi.nlm.nih.gov/geo/query/acc.cgi?acc=GSE225642].
