# Supplementary material for: Extracellular matrix stiffness modulates angiogenic properties of the retinal pigment epithelium
Source: Sci Rep. 2025 Nov 18;15:40349. doi: 10.1038/s41598-025-27140-4 (PMC12627602; doi:10.1038/s41598-025-27140-4)
Supplement: Supplementary file 1 — Supplementary Material 1 [file 41598_2025_27140_MOESM1_ESM.pdf]

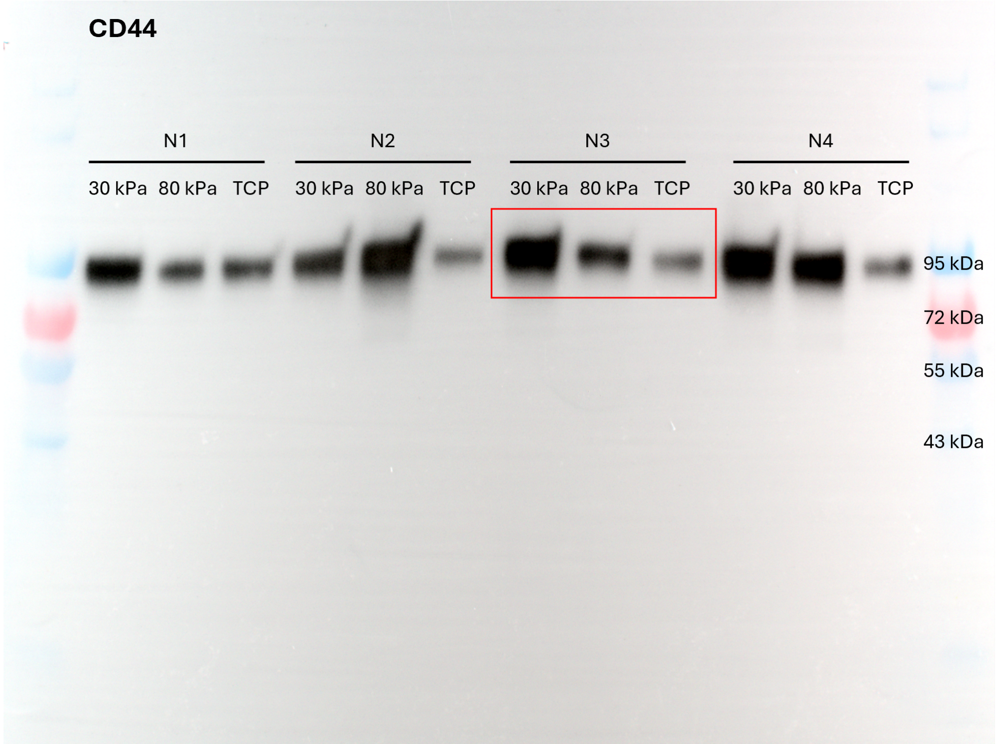


**Figure S1.** Stiffness-dependent expression of CD44 in ARPE-19 cells (full WB) with n=4 and the three lanes of interest from left to right representing 30 kPa, 80 kPa and TCP from the set shown in Figure 2A (red rectangle).

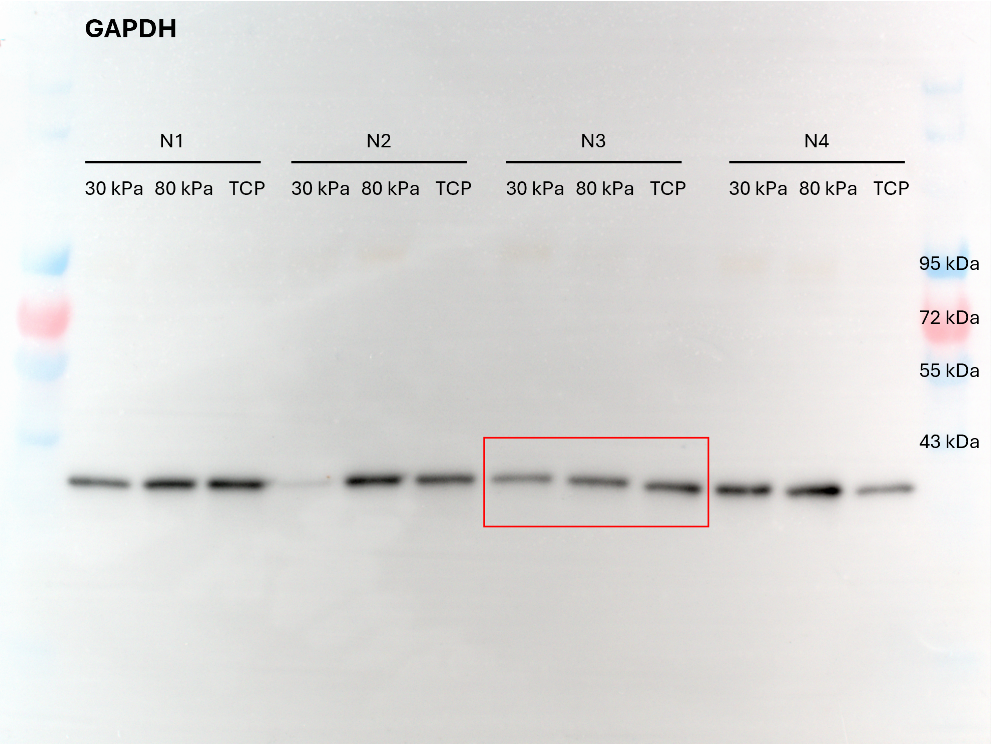


**Figure S2.** Loading control GAPDH in ARPE-19 cells (full WB) with n=4 and the three lanes of interest from left to right representing 30 kPa, 80 kPa and TCP from the set shown in Figure 2A (red rectangle).
